# ToF-SIMS Reveals Metformin-Driven Restoration of Hepatic Lipid and Amino Acid Profiles in a Type 2 Diabetes Rat Model

**DOI:** 10.3390/ijms27010105

**Published:** 2025-12-22

**Authors:** Magdalena E. Skalska, Michalina Kaźmierczak, Marcela Capcarova, Anna Kalafova, Klaudia Jaszcza, Dorota Wojtysiak

**Affiliations:** 1 Department of Medical Physics, M. Smoluchowski Institute of Physics, Faculty of Physics, Astronomy and Applied Computer Science, Jagiellonian University, 30-348 Krakow, Poland; michalina.kazmierczak@student.uj.edu.pl; 2 Centre for Theranostics, Jagiellonian University, 31-501 Krakow, Poland; 3 Institute of Applied Biology, Faculty of Biotechnology and Food Sciences, Slovak University of Agriculture in Nitra, 949 76 Nitra, Slovakiaanna.kalafova@uniag.sk (A.K.); 4 Department of Animal Physiology and Endocrinology, University of Agriculture in Krakow, Al. Mickiewicza 24/28, 30-059 Krakow, Poland; klaudia.jaszcza@urk.edu.pl; 5 Department of Genetics, Animal Breeding and Ethology, University of Agriculture in Krakow, Al. Mickiewicza 24/28, 30-059 Krakow, Poland

**Keywords:** time-of-flight secondary ion mass spectrometry, lipidomics, diabetes mellitus, liver metabolism, metformin

## Abstract

Diabetes mellitus profoundly disturbs hepatic metabolism by impairing lipid and amino acid homeostasis, yet spatially resolved molecular evidence of these alterations remains limited. This study employed Time-of-Flight Secondary Ion Mass Spectrometry (ToF-SIMS) to visualise and quantify metabolic remodelling in rat liver under diabetic conditions and following metformin treatment. Liver cryosections from lean controls (LEAN), diabetic rats (P1), and metformin-treated diabetic rats (P2) were analysed in the negative ion mode, and all spectra were normalised to total ion counts. One-way ANOVA with false discovery rate (FDR) correction identified 43 lipid-related and 20 amino acid-related ions with significant group differences. Diabetic livers exhibited a marked depletion of phospholipid- and fatty acid-related ions (e.g., *m*/*z* 241.04, 281.25, 536.38) accompanied by increased ceramide fragments (*m*/*z* 805–806), indicating lipotoxic remodelling and mitochondrial stress. Simultaneously, aromatic and neutral amino acids such as phenylalanine, tyrosine, and glutamine were reduced, while small acidic fragments were elevated, consistent with enhanced proteolysis and gluconeogenic flux. Metformin administration partially restored both lipid and amino acid profiles toward the control phenotype. Hierarchical clustering and spatial ion maps revealed distinct group separation and partial normalisation of hepatic molecular patterns. These results demonstrate that ToF-SIMS provides label-free, spatially resolved insights into diabetes-induced metabolic disturbances and metformin-driven hepatoprotection.

## 1. Introduction

Diabetes mellitus is a chronic metabolic disorder characterised by sustained hyperglycaemia resulting from impaired insulin secretion or insulin resistance [1,2]. The liver plays a pivotal role in maintaining glucose and lipid homeostasis, and therefore, it is one of the main organs affected by diabetes-associated metabolic dysregulation [3].

In the diabetic state, increased gluconeogenesis, enhanced lipogenesis, and mitochondrial dysfunction contribute to lipid accumulation, oxidative stress, and inflammation within hepatocytes, leading to hepatic steatosis and progressive metabolic impairment [4].

Metformin, the most widely prescribed oral antidiabetic drug, remains the first-line therapy for type 2 diabetes due to its efficacy, safety, and broad pleiotropic effects beyond glycaemic control [5,6]. Its primary mechanism of action involves inhibition of hepatic glucose production by modulating mitochondrial complex I and activating AMP-activated protein kinase (AMPK) [7]. In addition to improving insulin sensitivity, metformin influences lipid metabolism by reducing de novo lipogenesis, enhancing β-oxidation, and regulating phospholipid and fatty acid turnover [8].

Despite extensive clinical use, the precise molecular and metabolic consequences of metformin treatment in diabetic liver tissue remain incompletely characterised, particularly with respect to spatially resolved biochemical changes [5,8]. Conventional metabolomic and lipidomic methods based on solvent extraction provide detailed compositional information but obscure the tissue’s spatial organisation and microdomain heterogeneity. Time-of-Flight Secondary Ion Mass Spectrometry (ToF-SIMS) offers a complementary, label-free approach that enables simultaneous detection of lipids, amino acids, and other metabolites directly from tissue surfaces with submicrometre spatial resolution [9,10]. The technique provides unique insight into the molecular architecture of biological samples, capturing both compositional and localisation differences that are often lost in bulk analysis [11,12]. Recent studies on ToF-SIMS have demonstrated its applicability in mapping metabolic signatures in neurodegeneration, cancer, and diabetic complications [13,14]. In this study, we utilised ToF-SIMS in the negative ion mode to explore metabolomic changes in the livers of diabetic rats and to assess the impact of metformin treatment on hepatic molecular composition.

## 2. Results and Discussion

The overall molecular composition of liver tissue was first examined by assessing the class-level contributions of amino acid- and lipid-related ions detected in the negative ion mode (Table 1). The ToF-SIMS method used in this study provides highly surface-sensitive molecular information, probing only the outermost layers of liver cryosections. In the rat liver, macroscopically, four lobes can be distinguished: the larger right lobe and left lobe, and the smaller caudate lobe and quadrate lobe. The microstructure of the entire liver is homogeneous; it is composed of thousands of hexagonal hepatic lobules. Within each lobule are rows of hepatocytes (liver cells) separated by blood-filled capillaries (sinusoids) and bile canaliculi. Liver function is also independent of the lobe. Therefore, we can assume that the changes we observed affect not only the left lobe of the liver but also the entire organ.

In the diabetic group (P1), the relative abundance of amino acid-derived ions was significantly increased, whereas lipid-related peaks were markedly reduced compared with the lean controls (LEAN). This shift indicates enhanced proteolysis and impaired lipid turnover under hyperglycaemic conditions. Administration of metformin (P2) partially restored the lipid fraction while reducing the amino acid-related signal intensity, suggesting a normalisation of hepatic metabolic balance.

Liver cryosections from the LEAN, P1, and P2 groups were mounted on silicon wafers and analysed in the negative ion mode. Data processing included normalisation to total ion counts (TICs), statistical testing using one-way ANOVA with false discovery rate (FDR) correction, volcano and clustering analyses, and spatial visualisation of selected ions. This tiered analytical approach, progressing from global compositional profiling to feature-level statistics and spatial mapping, revealed distinct metabolic alterations associated with diabetes, including the depletion of phospholipids and aromatic amino acids and the accumulation of free fatty acids and small acidic fragments. These changes were partially normalised following metformin treatment, highlighting its corrective effect on hepatic lipid and amino acid metabolism.

### 2.1. Lipidomic Alterations and Partial Restoration by Metformin

**Figure 1:**
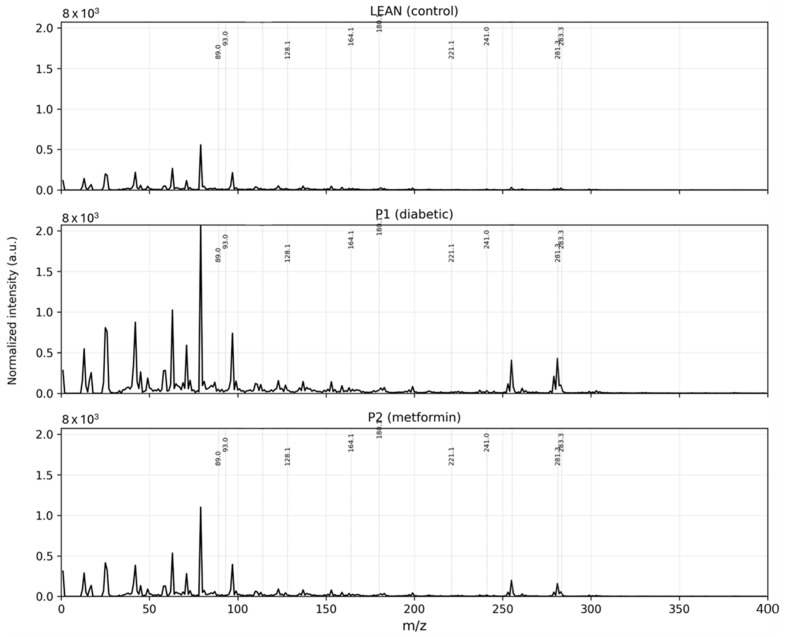
Averaged negative ion ToF-SIMS spectra of liver tissue from LEAN (control), P1 (diabetic), and P2 (metformin-treated) rats. Distinct group-dependent molecular patterns are visible across the 0–400 *m*/*z* range. Diabetic spectra (P1) show increased intensities of small acidic fragments (e.g., *m*/*z* 89.03, 90.03, 131.0) and a marked reduction in phospholipid- and fatty acid-related ions (e.g., *m*/*z* 241.0, 253.2, 281.27, 283.28) relative to LEAN. Metformin treatment (P2) results in intermediate profiles, with partial restoration of characteristic lipid and amino acid signals toward control levels. Annotated *m*/*z* values represent key diagnostic ions discussed in the text.

Representative negative ion spectra acquired from LEAN, P1, and P2 liver tissues exhibited reproducible, group-specific molecular profiles (Figure 1). The low-mass range (*m*/*z*, mass-to-charge ratio, 40–200) was dominated by amino acid fragments (e.g., *m*/*z* 89—Asp/Asn; *m*/*z* 114—Pro-related), whereas the higher range (*m*/*z* 200–900) was characterised by lipid-derived ions, including fatty acids (*m*/*z* 255, 281), phospholipid fragments (*m*/*z* 241, 536), and ceramide-related species (*m*/*z* 805).

**Figure 2:**
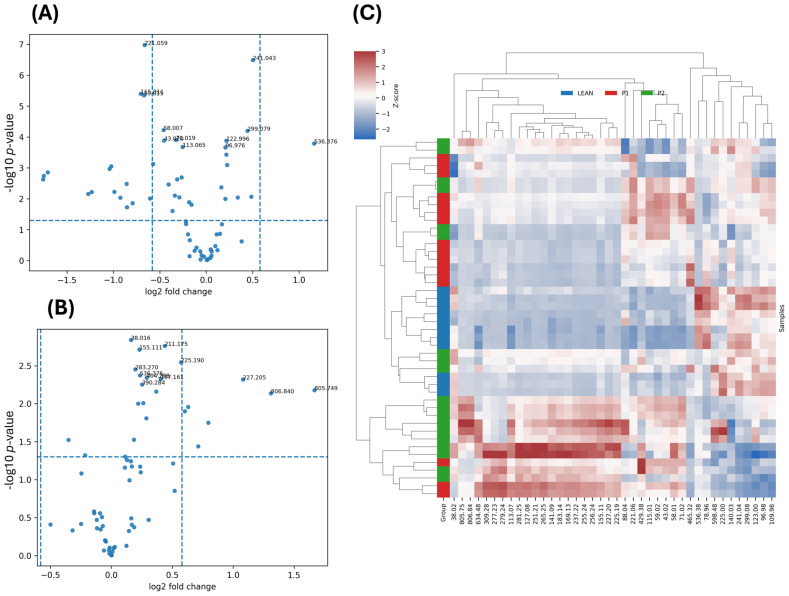
Negative ion ToF-SIMS lipid analysis of liver tissue. (**A**) Volcano plot for the LEAN vs. P1 comparison showing log_2_ fold change (x-axis) versus –log_10_(*p*-value) (y-axis). Ions exceeding the thresholds of |log_2_FC| > 0.6 and *p* < 0.05 are annotated; negative fold changes correspond to diabetes-associated decreases. (**B**) Volcano plot for the P2 vs. P1 comparison illustrating ions that shift toward LEAN levels following metformin treatment. (**C**) Hierarchical clustering heatmap (Z-scored intensities) of the 40 most significant lipid ions, demonstrating clear separation between LEAN and P1 groups and intermediate clustering of P2 samples, consistent with partial restoration of lipid signatures under metformin.

**Figure 3:**
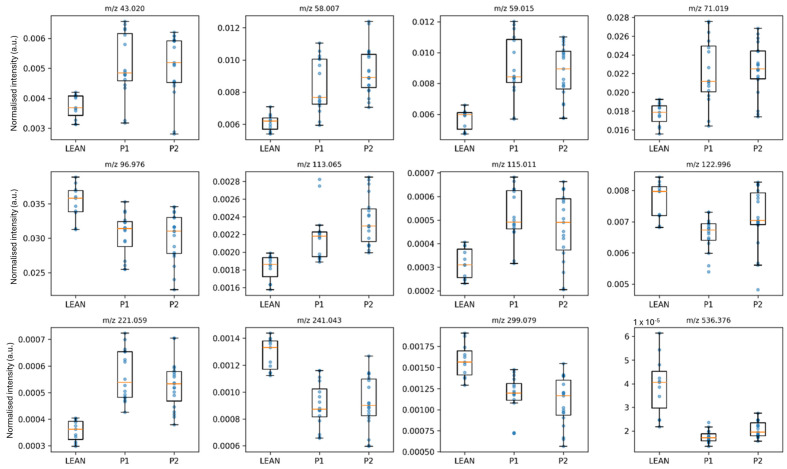
Representative boxplots illustrating the distribution of normalised intensities (a.u.) for lipid-related ions detected in rat liver tissues from LEAN (control), P1 (diabetic control), and P2 (metformin-treated) groups. Each boxplot represents the median, interquartile range, and individual data points for each group. The selected ions (e.g., phosphatidylinositol (PI) headgroup fragment [*m*/*z* 241.043], oleate (FA 18:1) [*m*/*z* 281.253], phosphatidylethanolamine (PE) species [*m*/*z* 536.376], ceramide fragment [*m*/*z* 805.749]) were chosen based on significant differences identified by ANOVA followed by FDR correction (*q* < 0.05) and biological relevance.

Based on pairwise contrasts, ions were categorised as Up vs. P1, Down vs. P1, or Mixed vs. P1. These features were visualised using volcano and box plots (Figure 2 and Figure 3), and the statistically significant ions are summarised in Tables S1 and S2 and in the Supplementary Materials.

The averaged negative ion spectra demonstrated clear group-dependent molecular signatures across the LEAN, P1, and P2 samples, as shown in Figure 1. P1 exhibited a characteristic suppression of major lipid-derived ions, including phosphatidylinositol fragments (*m*/*z* 241.04), fatty acid anions (*m*/*z* 255.23, 281.25), and phosphatidylethanolamine fragments (*m*/*z* 536.38). This global reduction in lipid-related signals is consistent with diabetes-associated impairment of membrane remodelling.

In contrast, the LEAN group displayed the highest intensities for these lipid species, forming a profile typical of healthy hepatic metabolism. The P2 group showed intermediate intensities: lipid peaks were markedly higher than in P1 yet did not fully reach LEAN levels, illustrating partial biochemical restoration. Differences were also evident in the amino acid region of the spectra (*m*/*z* 40–200). The P1 spectra exhibited elevated signals for small acidic fragments (e.g., *m*/*z* 89.03, 93.05, 114.03), reflecting increased proteolysis and enhanced amino acid-driven gluconeogenesis, whereas the LEAN spectra showed higher levels of intact amino acid anions such as phenylalanine (*m*/*z* 164.07) and tyrosine (*m*/*z* 180.05). Again, P2 profiles shifted toward LEAN, indicating recovery of amino acid turnover under metformin.

A total of 43 lipid-related ions showed significant group effects according to ANOVA with FDR correction (*q* < 0.05; Table 2; full dataset in Table S1). The most discriminant ions corresponded to fragments of phosphatidylinositol (*m*/*z* 241.04) and phosphatidylethanolamine (*m*/*z* 536.38) as well as free fatty acids (*m*/*z* 281.25, 283.27).

Based on pairwise fold-change patterns relative to the diabetic control (P1), the significant ions were grouped as Up vs. P1 (increased relative to P1, reflecting diabetes-associated depletion and metformin-driven restoration), Down vs. P1 (decreased in P1, reflecting lipid accumulation under hyperglycaemia), and Mixed vs. P1 (divergent or bidirectional behaviour across groups). Among the 43 features, 10 were classified as Up vs. P1, 4 as Down vs. P1, and 29 as Mixed vs. P1.

The most prominent Up vs. P1 ions included *m*/*z* 536.376 (phosphatidylinositol fragment; *q* = 1.1 × 10^−8^) and *m*/*z* 241.043 (palmitoleic acid fragment; *q* = 1.6 × 10^−5^), both reduced in diabetic livers and partially restored upon metformin treatment. Similar restorative trends were noted for stearic acid (*m*/*z* 283.270) and phospholipid fragments (*m*/*z* 404.288 and *m*/*z* 402.267), consistent with partial normalisation of fatty acid and glycerophospholipid pools.

Conversely, Down vs. P1 ions such as *m*/*z* 221.059 (myristic acid fragment), *m*/*z* 115.011 (aspartic acid ion), and *m*/*z* 465.318 (phosphatidic acid fragment) were elevated in diabetes and decreased toward control levels under metformin treatment, suggesting correction of diabetes-induced lipid accumulation. The largest subset displayed Mixed vs. P1 behaviour (e.g., *m*/*z* 281.253 for oleic/linoleic acid and *m*/*z* 805.749 and 806.840 for phosphatidylinositol-related species), reflecting complex hepatic lipid remodelling and incomplete compensation following metformin therapy.

To provide a quantitative context, we calculated the percentage of the LEAN–P1 difference recovered in the metformin-treated group (P2) for representative ions. For key lipid-related ions that were depleted in diabetes and increased under metformin, the recovery ranged from modest to substantial. The phosphatidylinositol fragment at *m*/*z* 241.043 showed a recovery of ~4.8%, whereas the phosphatidylethanolamine-related ion at *m*/*z* 536.376 recovered ~14.3% of the LEAN–P1 difference. Among ions elevated in diabetes and decreased by metformin, the phosphatidic acid fragment at *m*/*z* 465.318 exhibited ~67.3% recovery, while the myristic acid-related fragment (*m*/*z* 221.059) and the aspartic acid ion (*m*/*z* 115.011) showed ~16.7% and ~24.0% recovery, respectively.

Volcano plots (Figure 2A,B) visualised a broad depletion of phospholipid and fatty acid signals in P1 and their partial normalisation in P2. Representative boxplots (Figure 3) confirmed this recovery, with P2 distributions shifting closer to LEAN values. In the LEAN vs. P1 comparison, most ions displayed reduced intensities in diabetes (e.g., *m*/*z* 241.043 PI-fragment, *m*/*z* 281.253 oleate, *m*/*z* 536.376 PE species), whereas ceramide- and short acyl-related peaks (*m*/*z* 805.749, *m*/*z* 221.059) were increased, indicating altered sphingolipid metabolism.

The hierarchical clustering heatmap (Figure 2C) further supported these findings, clearly discriminating between LEAN, P1, and P2 samples. The diabetic cluster (P1) was characterised by decreased intensities of phospholipid and fatty acid fragments (*m*/*z* 241.04, 281.25, 402.27, 536.38), typical for oxidative stress and impaired lipid turnover. Metformin-treated samples (P2) showed an intermediate clustering pattern, approaching LEAN profiles, indicating partial restoration of membrane lipid composition and β-oxidation efficiency. In contrast, high-mass ions (*m*/*z* 700–810) tentatively assigned to ceramide or sphingomyelin species remained elevated, suggesting that metformin alleviates—but does not completely reverse—sphingolipid dysregulation [15]. Similar signatures have been reported in human and experimental models of diabetes, in which ceramide accumulation contributes to impaired insulin signalling and oxidative stress, while decreased phospholipid remodelling reflects disrupted membrane turnover [16,17,18].

Metformin treatment led to partial normalisation of these lipid features, which aligns with activation of AMP-activated protein kinase (AMPK), inhibition of acetyl-CoA carboxylase, and suppression of sterol regulatory element-binding protein (SREBP)-dependent lipogenesis [19,20,21]. Restoration of phosphatidylethanolamine and phosphatidylinositol fragments toward control levels is consistent with improved mitochondrial β-oxidation and balanced phospholipid biosynthesis under metformin therapy [22].

### 2.2. Amino Acid-Related Alterations

Twenty amino acid-related ions were found to differ significantly among groups after FDR correction (*q* < 0.05; Table 3; full dataset in Table S2). Decreased signals were observed for *m*/*z* 164.07 (phenylalanine), *m*/*z* 180.05 (tyrosine), and *m*/*z* 128.07 (glutamine/glutamate) in diabetic livers, indicating suppression of amino acid turnover under hyperglycaemia. Metformin treatment (P2) increased the intensity of these ions toward LEAN values. 

Relative to the diabetic control (P1), 3 ions were classified as Up vs. P1 (lower in P1 and partially restored with metformin), 6 as Down vs. P1 (higher in P1 and decreased toward LEAN in P2), and 11 displayed Mixed vs. P1 patterns. Among the top signals, *m*/*z* 180.051 and *m*/*z* 164.071, consistent with tyrosine [M–H]^−^ and phenylalanine [M–H]^−^, respectively, were markedly reduced in P1 and increased under metformin (*q* = 3.6 × 10^−6^ and 6.9 × 10^−6^; Table 3). In contrast, small acidic fragments such as *m*/*z* 93.046 and *m*/*z* 114.027 (fragment-like ions) and lactate [M–H]^−^, *m*/*z* 89.025 were elevated in P1 and decreased in P2, indicating partial correction of diabetes-associated accumulation (*q* ≤ 5 × 10^−3^; Table 3). A larger subset exhibited mixed versus P1 behaviour (e.g., *m*/*z* 128.067, 153.017, 209.033), suggesting heterogeneous or overcompensated responses under metformin therapy.

Amino acid-related ions displayed comparable patterns: tyrosine (*m*/*z* 180.05) and phenylalanine (*m*/*z* 164.07) recovered ~10.9% and ~23.1% of the distance between P1 and LEAN, respectively, whereas small acidic fragments associated with lactate/aspartate and related species (*m*/*z* 93.05, 114.03, 89.03, 122.01) recovered ~47.6%, ~30.3%, ~19.9%, and ~38.3%.

Collectively, the amino acid profile mirrors the lipidomic findings. Diabetes depletes aromatic amino acids (Phe, Tyr) and elevates small acidic species, whereas metformin tends to normalise these signals toward LEAN, albeit not uniformly across all ions (Table 3 and Table S2).

**Figure 4:**
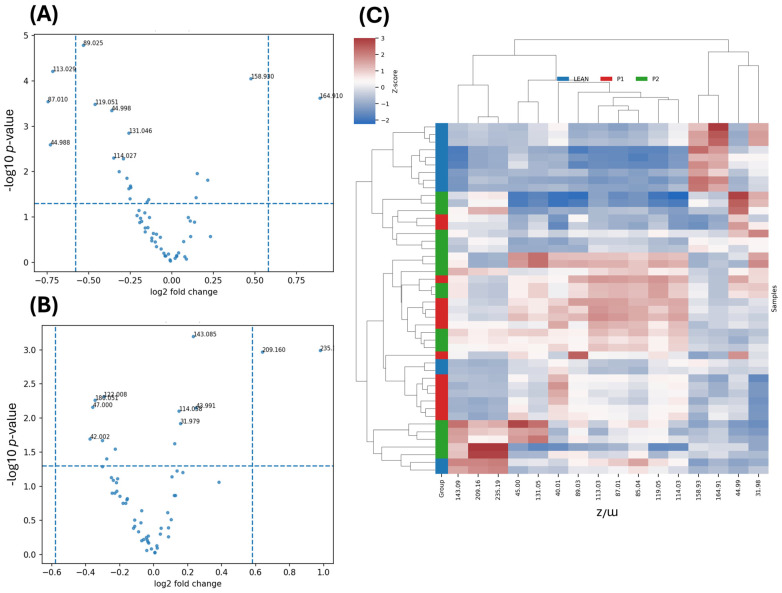
Positive ion ToF-SIMS analysis of liver tissue. (**A**) Volcano plot for the LEAN vs. P1 comparison showing log_2_ fold change versus –log_10_(*p*-value). Several choline-containing fragments and phospholipid-derived ions (e.g., *m*/*z* 86.10, 104.11, 113.05, 158.98) are significantly decreased in diabetic liver, reflecting impaired membrane lipid composition under hyperglycaemia. (**B**) Volcano plot for the P2 vs. P1 comparison indicating ions that shift toward LEAN levels following metformin treatment; significant features (*p* < 0.05, |log_2_FC| > 0.6) demonstrate partial restoration of positive ion lipid signatures. (**C**) Hierarchical clustering heatmap (Z-scored intensities) of the most discriminatory positive ion signals, showing clear separation between LEAN and P1 groups and intermediate clustering of the P2 samples, consistent with metformin-driven correction of diabetes-associated molecular alterations.

Volcano and box plots (Figure 4A,B) revealed that aromatic and branched-chain amino acids were depleted in diabetes, whereas small acidic fragments (*m*/*z* 89, 93, 114) were elevated, reflecting enhanced proteolysis and amino acid-driven gluconeogenesis. Metformin reversed several of these changes, particularly the restoration of *m*/*z* 164.07 (Phe) and *m*/*z* 180.05 (Tyr), suggesting recovery of hepatic amino acid metabolism.

The clustering of amino acid-derived ions (Figure 4C) revealed distinct metabolic grouping across the three experimental conditions. P1 (diabetic) samples clustered separately, showing lower Z-scores (blue) for the majority of amino acid fragments, including *m*/*z* 89.03 (aspartate/asparagine), 93.05 (glycine/alanine), 114.03 (proline), 128.07 (glutamine/glutamate), and 164.07 (arginine). This pattern consists of reduced amino acid turnover and impaired nitrogen metabolism in the diabetic liver.

Conversely, metformin treatment (P2) shifted the clustering pattern closer to that in LEAN controls, accompanied by higher relative intensities for several of these ions, indicating recovery of amino acid catabolism and protein degradation pathways. The hierarchical dendrogram shows a clear grouping of LEAN and P2 samples, supporting the observation that metformin restores a more physiological amino acid profile.

**Figure 5:**
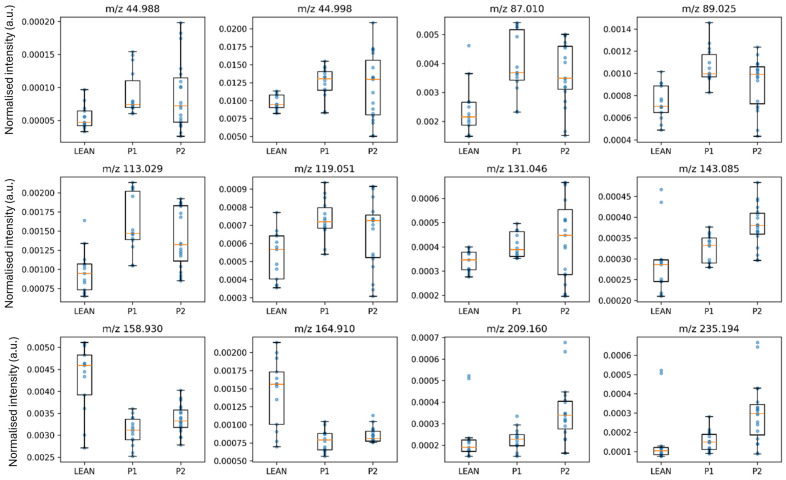
Boxplots showing normalised signal intensities (a.u.) for representative amino acid-related ions across LEAN (control), P1 (diabetic control), and P2 (metformin-treated) groups. Statistical significance was determined by one-way ANOVA followed by Tukey’s post hoc test with FDR correction (*q* < 0.05). The selected ions include *m*/*z* 89.025 (aspartate/asparagine fragment), *m*/*z* 93.046 (glycine/alanine fragment), *m*/*z* 114.027 (proline-related ion), *m*/*z* 128.067 (glutamine/glutamate-related ion), and *m*/*z* 164.071 (arginine-related ion), representing key amino acid species associated with energy and nitrogen metabolism.

Notably, the strongest recovery effects are observed at *m*/*z* 89.03 and 128.07, suggesting enhanced transamination and TCA-linked amino acid fluxes upon metformin administration. In summary, the heatmap confirms that diabetes suppresses hepatic amino acid-related signals, whereas metformin partly re-establishes metabolic balance, reflecting improved mitochondrial and nitrogen-handling efficiency. The decreased levels of aromatic and neutral amino acids—phenylalanine, tyrosine, and glutamine/glutamate—indicate suppressed protein synthesis and TCA-linked anaplerotic fluxes, consistent with previous metabolomic observations in insulin-resistant states [23,24].Volcano and box plots (Figure 5) revealed that aromatic and branched-chain amino acids were depleted in diabetes, whereas small acidic fragments (*m*/*z* 89, 93, 114) were elevated.

The amino acid profile also reflected a diabetic metabolic shift. Decreased intensities of aromatic and neutral amino acids (tyrosine, phenylalanine, glutamine) and increased levels of small acidic fragments (lactate, aspartate) were observed, indicating enhanced protein breakdown and amino acid-driven gluconeogenesis [25,26]. Metformin partly reversed these effects, in line with its reported ability to suppress hepatic gluconeogenesis by modulating mitochondrial redox balance and amino acid fluxes [24,27].

### 2.3. Spatial Ion Distributions

**Figure 6:**
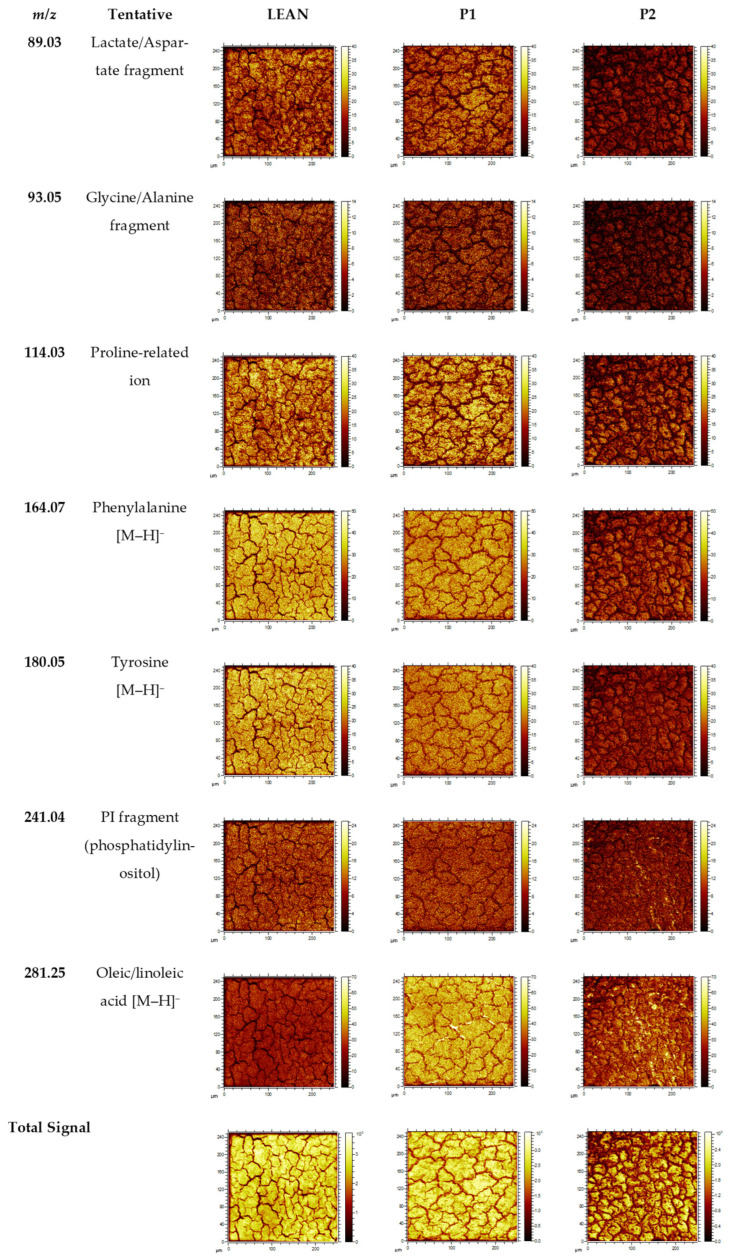
Two-dimensional ToF-SIMS ion maps showing representative lipid- and amino acid-related ions detected in liver sections from control (LEAN), diabetic (P1), and metformin-treated (P2) rats. Rows correspond to selected *m*/*z* values characteristic of phospholipids and fatty acids (e.g., *m*/*z* 241.04—PI fragment, *m*/*z* 281.25—oleic/linoleic acid) and amino acids (e.g., *m*/*z* 89.03—aspartate/lactate, *m*/*z* 114.03—proline, *m*/*z* 164.07—phenylalanine, *m*/*z* 180.05—tyrosine). All images were normalised to total ion counts (TICs) and visualised with fixed-intensity scales within each ion row to ensure comparability between groups.

To further visualise the spatial context of the molecular alterations, two-dimensional ToF-SIMS ion maps were generated for representative lipid- and amino acid-related ions (Figure 6). Distinct spatial patterns were observed among the three experimental groups. In P1, the distributions of phosphatidylinositol (*m*/*z* 241.04) and oleic/linoleic acid (*m*/*z* 281.25) were markedly reduced, reflecting lipid depletion and altered membrane composition under hyperglycaemic stress. Concurrently, amino acid fragments such as aspartate/lactate (*m*/*z* 89.03) and proline (*m*/*z* 114.03) displayed higher relative intensities, consistent with increased proteolysis and disturbed amino acid turnover. Conversely, signals of aromatic amino acids, notably phenylalanine (*m*/*z* 164.07) and tyrosine (*m*/*z* 180.05), were substantially diminished in the diabetic group, indicating impaired protein synthesis and oxidative degradation.

Beyond lipids, ToF-SIMS imaging revealed pronounced perturbations in amino acid-related ions in diabetic livers. The decreased levels of aromatic and neutral amino acids, like phenylalanine, tyrosine, and glutamine/glutamate, indicate suppressed protein synthesis and TCA-linked anaplerotic fluxes, consistent with previous metabolomic observations in insulin-resistant states [26,27]. Conversely, elevated signals of small acidic fragments (aspartate, lactate, proline) suggest increased proteolysis and amino acid-driven gluconeogenesis, pathways that fuel hepatic glucose production in diabetes [1,3,19,26].

Although ToF-SIMS does not allow routine MS/MS fragmentation in static imaging mode, we applied several forms of orthogonal validation, including reproducible detection across technical replicates, alignment with known SIMS fragmentation patterns, spatial localisation consistent with tissue structure, and concordance with previously published LC-MS/MS and NMR metabolomics in diabetic liver [15,16,17,25,26]. These complementary validation layers strengthen confidence in the structural assignments despite the lack of direct tandem MS.

Several of the lipid and amino acid alterations observed in the ToF-SIMS spectra are consistent with the known metabolic effects of metformin. Restoration of phosphatidylinositol- and phosphatidylethanolamine-derived fragments, together with the partial recovery of long-chain fatty acids, aligns with reports that metformin activates AMPK-dependent pathways, leading to inhibition of ACC1/ACC2 and improved mitochondrial fatty acid oxidation [5,6,22]. Likewise, the reversal of diabetes-associated elevations in lactate/aspartate (*m*/*z* 89.03) and other acidic fragments corresponds with the drug’s ability to modulate redox state and suppress amino acid-driven gluconeogenesis [23,24,25]. Although ToF-SIMS does not directly measure enzyme activity, the observed molecular changes follow patterns characteristic of restored mitochondrial efficiency and nitrogen handling under metformin therapy.

## 3. Materials and Methods

### 3.1. Animals and Experimental Design

All experimental procedures were approved by the Ethics Committee and the State Veterinary and Food Administration of the Slovak Republic (approval number 5159-4/2024-220) and were conducted in accordance with Directive 2010/63/EU on the protection of animals used for scientific purposes. Male Zucker Diabetic Fatty (ZDF) rats (fa/fa) and their lean littermates (FA/FA or Fa/fa) aged 12 weeks were obtained from the Dobrá Voda accredited breeding facility (Slovak Republic, SK CH 20021). Animals were housed in standard polycarbonate cages with woodchip bedding under controlled environmental conditions (21 ± 1 °C, 50 ± 10% relative humidity, 12 h light/dark cycle) with free access to tap water and standard chow (KKZ-P/M, complete feed mixture for rats and mice, registration No. 6147, Dobrá Voda, Slovakia). After a one-week acclimatisation period, animals were randomly divided into three experimental groups: LEAN (control) (n = 4), consisting of non-diabetic rats without the fa gene mutation; P1 (diabetic control) (n = 8), comprising ZDF rats with spontaneous type 2 diabetes receiving distilled water; and P2 (metformin-treated diabetic) (n = 8), which included ZDF rats treated with metformin using a gastric probe.

Unlike in streptozotocin (STZ)-induced models of type 1 diabetes, the ZDF rats used in this study develop spontaneous type 2 diabetes due to a mutation in the leptin receptor gene (fa), leading to progressive obesity, insulin resistance, and hyperglycaemia as previously described by Kalafová et al. and Capcarová et al. [23,28].

#### 3.1.1. Treatment Protocol

Metformin (1,1-dimethylbiguanide hydrochloride, Sigma-Aldrich, St. Louis, MO, USA) was freshly dissolved in distilled water immediately before administration and was given by oral gavage once daily (including weekends) at a dose of 150 mg/kg b.w. throughout the 12-week experimental period, as described previously [29]. The LEAN and diabetic control (P1) groups received an equivalent volume of distilled water.

The metformin dose (150 mg/kg body weight/day) was selected based on previous studies conducted in the same research unit (Slovak University of Agriculture, Nitra) and on published work employing the ZDF model, in which this dose consistently produces physiologically relevant plasma exposure and reproducible metabolic effects. The same dosing regimen has been used in earlier experiments performed by Dupak et al., ensuring consistency with previous experiments and enabling reliable comparison across studies within the same research programme.

#### 3.1.2. Sample Collection

The unequal group sizes reflect the Mendelian distribution of genotypes within the Zucker Diabetic Fatty (ZDF) colony, where LEAN (Fa/Fa or Fa/fa) animals occur less frequently than diabetic fa/fa rats. Similar sample size ratios have been reported previously in metabolic and histological studies using ZDF rats [23,28,29]. To minimise the impact of reduced control group size on statistical power, three technical replicates per sample were acquired.

**Figure 7:**
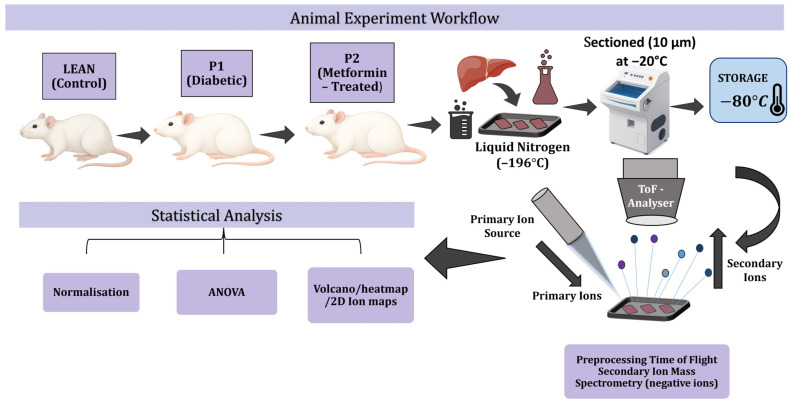
Schematic overview of experimental design. Liver tissues were collected from control (LEAN), diabetic (P1), and metformin-treated (P2) rats. Cryosections (10 µm) were mounted on silicon wafers and analysed using ToF-SIMS in the negative ion mode. Data processing included normalisation to total ion counts, statistical testing (ANOVA with FDR correction), volcano and clustering analyses, and visualisation of spatial ion distributions.

At the end of the experimental period, animals were anaesthetised and sacrificed by cardiac puncture. The liver was immediately excised, rinsed with 0.9% NaCl, and gently blotted dry, and a fragment of the left hepatic lobe was consistently collected from each animal. This sampling strategy was adopted to minimise anatomical variability. The tissue fragments were snap-frozen in liquid nitrogen and stored at –80 °C until further ToF-SIMS analysis. The experimental workflow of the ToF-SIMS analysis is illustrated in Figure 7.

### 3.2. Sample Preparation for ToF-SIMS

Frozen liver samples were sectioned (10 µm thick) at −20 °C using a cryostat (Slee MEV, Mainz, Germany) and mounted on cleaned (ultrasonication in toluene and ethanol) silicon wafers (cat. No. 647780, Sigma Aldrich, St. Louis, MO, USA). Sections were desiccated under vacuum and stored in sealed Petri dishes to prevent contamination. Before analysis, samples were equilibrated to room temperature in a dry nitrogen atmosphere. The group size imbalance reflects Mendelian inheritance in the ZDF colony, where LEAN (Fa/Fa) animals occur less frequently. Similar group sizes have been reported in previous ZDF studies [23,28,29]. To compensate for reduced power, three technical replicates per sample were acquired, and FDR correction was applied.

### 3.3. ToF-SIMS Measurements

ToF-SIMS analyses were performed on a ToF-SIMS 5 instrument (ION-TOF GmbH, Münster, Germany) equipped with a Bi_3_^+^ liquid metal ion gun operated at 30 keV. Measurements were conducted under ultra-high vacuum (1.7 × 10^−9^ mbar). The primary ion beam was incident at 45° relative to the surface. In static SIMS mode, the total ion fluence was maintained below 1 × 10^12^ ions/cm^2^ to prevent surface damage. Additional fast-imaging acquisitions were performed to visualise ion distributions with higher spatial throughput. The typical lateral spatial resolution achieved was approximately 2–5 μm, with a mass resolving power of m/Δm ≈ 4000 at *m*/*z* 29.

Each liver section was analysed in both positive and negative ion modes, with three replicates per mode, over an area of 250 × 250 µm^2^ (128 × 128 pixels). Charge compensation was achieved through low-energy electron flooding. Spectra were acquired over the range *m*/*z* 0–911, where *m*/*z* denotes the mass-to-charge ratio of detected ions, and then internally calibrated using reference negative ions: H^−^, C^−^, CH^−^, C_2_^−^, C_3_^−^ ensuring mass accuracy better than 300 ppm across the acquired mass range. As ToF-SIMS is inherently semi-quantitative, relative signal intensities were compared following normalisation to total ion counts (TICs), which is the standard approach for comparative molecular imaging in biological tissues. Data acquisition and processing were conducted using SurfaceLab 7.2 (ION-TOF GmbH). For quantitative analysis, central regions of interest (ROIs) measuring 150 × 150 µm^2^ were selected to minimise edge effects and charging artefacts.

### 3.4. Data Processing and Statistical Analysis

Normalised intensity data were exported to OriginPro 2020b (v. 9.8.0.200; OriginLab Corp., Northampton, MA, USA) for statistical processing. The distribution of each variable was verified using the Shapiro–Wilk test, and homogeneity of variances was assessed with Levene’s test. Data are presented as mean ± SEM. Group differences among LEAN, P1, and P2 were evaluated using one-way ANOVA, followed by Tukey’s post hoc test to assess pairwise comparisons. To control for multiple testing, the resulting *p*-values were additionally adjusted using the Benjamini–Hochberg false discovery rate (FDR) procedure. The resulting *q*-value represents the minimum FDR at which a given ion is considered significant and corresponds to the expected proportion of false positives among all features declared differentially expressed. In accordance with metabolomic and imaging mass spectrometry standards, ions with *q* < 0.05 were regarded as statistically significant.

For illustrative purposes, the degree of recovery in the metformin-treated group (P2) was expressed as a percentage of the difference between LEAN and P1, calculated as (I_P2 − I_P1)/(I_LEAN − I_P1) × 100, where I denotes TIC-normalised mean intensity for a given ion in a given group. Values of 0% and 100% correspond to no change relative to P1 and full convergence towards the LEAN level, respectively. These percentages should be interpreted as relative semi-quantitative measures derived from ToF-SIMS intensities rather than as absolute concentration changes.

Lipid- and amino acid-related ions were annotated using internal ToF-SIMS compound libraries and cross-referenced with the LIPID MAPS Structure Database (LMSD) for confirmation of putative assignments.

For spatial evaluation, ion images were visualised using fixed-intensity scales within each m/z channel to allow qualitative comparison between groups. Quantitative analysis was based on spectra extracted from a centrally positioned region of interest (ROI; 150 × 150 µm^2^), selected to minimise edge artefacts, surface defects, and beam-induced heterogeneity. ROI-averaged intensities were used for all statistical comparisons, as this approach is widely accepted in ToF-SIMS workflows and provides robust quantification without the confounding influence of local microtopography. Therefore, the ion maps included in this work serve as qualitative spatial illustrations supporting the group-dependent chemical differences detected in the ROI-based analysis.

## 4. Conclusions

In summary, the ToF-SIMS method enabled the direct visualisation of diabetes-induced metabolic remodelling in rat liver and its partial normalisation following metformin treatment. Negative ion spectra revealed that hyperglycaemia led to depletion of phospholipids and unsaturated fatty acids, accumulation of ceramide-related species, and suppression of amino acid turnover. Administration of metformin partially restored both lipid and amino acid profiles, resulting in a molecular pattern intermediate between diabetic and control states. The presented results demonstrate that ToF-SIMS offers a powerful platform for spatially resolved metabolic phenotyping, providing molecular-level evidence of metformin’s hepatoprotective action and its potential to rebalance disrupted metabolic networks in diabetes.

## Figures and Tables

**Table 1:** Relative contribution of amino acid- and lipid-related ions in rat liver ToF-SIMS spectra in negative ion mode.

	Amino Acids [%] (Mean ± SEM)	Lipids [%] (Mean ± SEM)
Lean	44.78 ± 1.58 ^a^	55.22 ± 1.58 ^b^
P1	47.42 ± 1.14 ^b^	52.57 ± 1.14 ^a^
P2	43.57 ± 1.42 ^a^	56.43 ± 1.42 ^b^

Lean—healthy control; P1—diabetic control; P2—metformin-treated diabetic rats; a, b—different superscript letters within each column indicate statistically significant differences (*p* < 0.05). Values are expressed as mean ± SEM.

**Table 2:** Statistically significant lipid-related ions detected in negative ion mode (LEAN, P1, P2). Data represent mean normalised intensities, log_2_ fold change relative to diabetic control (P1), and FDR-adjusted *q*-values.

*m*/*z*	Annotation	Trend vs. P1	LEAN	P1	P2	log_2_FC LEAN vs. P1	log_2_FC P2 vs. P1	*q* Value
536.376	PI frag. (phosphatidylinositol)	Up	3.9 × 10^−5^	1.8 × 10^−5^	2.1 × 10^−5^	1.16	0.24	1.07 × 10^−8^
241.043	FA frag. (palmitoleic acid)	Up	1.28 × 10^−3^	9.06 × 10^−4^	9.24 × 10^−4^	0.50	0.03	1.55 × 10^−5^
221.059	Myristic acid frag.	Down	3.56 × 10^−4^	5.65 × 10^−4^	5.30 × 10^−4^	−0.66	−0.09	7.08 × 10^−6^
281.253	Oleic/linoleic acid [M–H]^−^	Mixed	2.8 × 10^−3^	9.6 × 10^−3^	1.37 × 10^−2^	−1.75	0.52	2.6 × 10^−3^
283.270	Stearic acid [M–H]^−^	Up	4.35 × 10^−3^	4.21 × 10^−3^	4.82 × 10^−3^	0.05	0.20	8.2 × 10^−3^
404.288	Phospholipid frag.	Up	4.32 × 10^−5^	4.15 × 10^−5^	5.08 × 10^−5^	0.06	0.29	1.3 × 10^−2^
115.011	Aspartic acid	Down	3.18 × 10^−4^	5.18 × 10^−4^	4.70 × 10^−4^	−0.71	−0.14	9.4 × 10^−4^
465.318	Phosphatidic acid frag.	Down	9.14 × 10^−5^	1.36 × 10^−4^	1.06 × 10^−4^	−0.57	−0.35	6.3 × 10^−3^
805.749	PS/PI molecular ion	Mixed	2.97 × 10^−6^	5.89 × 10^−6^	1.87 × 10^−5^	−0.99	1.67	2.5 × 10^−3^
806.840	PS/PI adduct	Mixed	3.29 × 10^−6^	5.00 × 10^−6^	1.24 × 10^−5^	−0.61	1.31	2.6 × 10^−3^

LEAN—healthy control; P1—diabetic control; P2—metformin-treated diabetic rats. The *q*-value represents the false discovery rate (FDR)-adjusted *p*-value obtained using the Benjamini–Hochberg correction. It reflects the expected proportion of false positives among all features declared significant. Lower *q*-values indicate higher statistical confidence. In this study, ions with *q* < 0.05 were considered statistically significant.

**Table 3:** Statistically significant amino acid-related ions detected in negative ion mode (LEAN, P1, P2). Data represent mean normalised intensities, log_2_ fold-change values relative to diabetic control (P1), and FDR-adjusted *q*-values.

*m*/*z*	Annotation	Trend vs. P1	Mean LEAN	Mean P1	Mean P2	log2FC LEAN/P1	log2FC P2/P1	*q*
180.05	Tyrosine [M–H]^−^	Up	1.45 × 10^−3^	7.81 × 10^−4^	8.54 × 10^−4^	0.890	0.128	3.56 × 10^−6^
164.07	Phenylalanine [M–H]^−^	Up	4.30 × 10^−3^	3.09 × 10^−3^	3.37 × 10^−3^	0.476	0.124	6.87 × 10^−6^
93.05	Small acidic frag.	Down	7.40 × 10^−4^	1.07 × 10^−3^	9.13 × 10^−4^	−0.532	−0.228	2.99 × 10^−3^
114.03	Acidic AA frag.	Down	9.73 × 10^−4^	1.60 × 10^−3^	1.41 × 10^−3^	−0.717	−0.180	2.67 × 10^−3^
153.02	AA frag. (aromatic/amide)	Mixed	2.89 × 10^−4^	3.23 × 10^−4^	3.79 × 10^−4^	−0.161	0.232	2.67 × 10^−3^
128.07	Amino acid-related ion	Mixed	2.46 × 10^−3^	2.24 × 10^−3^	1.83 × 10^−3^	0.136	−0.294	4.82 × 10^−3^
89.03	Lactate [M–H]^−^	Down	2.38 × 10^−3^	3.99 × 10^−3^	3.67 × 10^−3^	−0.746	−0.120	4.41 × 10^−3^
209.03	Amino acid/peptidic fragment	Mixed	1.83 × 10^−3^	1.70 × 10^−3^	1.34 × 10^−3^	0.113	−0.345	1.06 × 10^−2^
40.01	Small frag. (C/O)	Mixed	5.29 × 10^−4^	5.85 × 10^−4^	6.59 × 10^−4^	−0.145	0.172	1.18 × 10^−2^
86.03	AA frag.	Mixed	2.36 × 10^−3^	2.89 × 10^−3^	2.90 × 10^−3^	−0.293	0.0057	1.20 × 10^−2^
87.01	AA frag.	Mixed	1.17 × 10^−3^	1.31 × 10^−3^	1.41 × 10^−3^	−0.164	0.107	2.50 × 10^−2^
122.01	Anion (AA/acid)	Down	5.33 × 10^−4^	7.34 × 10^−4^	6.57 × 10^−4^	−0.462	−0.159	2.37 × 10^−2^
42.00	Small fragment	Mixed	9.74 × 10^−3^	1.16 × 10^−2^	1.23 × 10^−2^	−0.253	0.0871	2.74 × 10^−2^
44.02	Small fragment	Mixed	5.46 × 10^−4^	5.71 × 10^−4^	6.79 × 10^−4^	−0.0661	0.250	1.64 × 10^−2^
65.01	Amino frag. (amine)	Mixed	4.52 × 10^−2^	3.90 × 10^−2^	3.86 × 10^−2^	0.214	−0.0151	1.64 × 10^−2^
49.01	Small anion	Down	6.27 × 10^−4^	7.57 × 10^−4^	5.90 × 10^−4^	−0.271	−0.359	4.70 × 10^−2^

LEAN—healthy control; P1—diabetic control; P2—metformin-treated diabetic rats. Ions with *q* < 0.05 were considered statistically significant.

## Data Availability

The original data presented in this study are openly available in RODBUK, Jagiellonian University in Krakow, at doi: 10.57903/UJ/GJPGY7.

## Supplementary Materials

The following supporting information can be downloaded at https://www.mdpi.com/article/10.3390/ijms27010105/s1.
