# Supplementary material for: ToF-SIMS Reveals Metformin-Driven Restoration of Hepatic Lipid and Amino Acid Profiles in a Type 2 Diabetes Rat Model
Source: Int J Mol Sci. 2025 Dec 22;27(1):105. doi: 10.3390/ijms27010105 (PMC12785574; doi:10.3390/ijms27010105)
Supplement: Supplementary file 1 [file ijms-27-00105-s001.zip › ijms-4016811-supplementary.pdf]

Table S1. List of statistically significant lipid-related ions detected in negative-ion mode (LEAN, P1, P2). Data represent mean normalised intensities, $\log_2$ fold changes relative to diabetic control (P1), and FDR-adjusted q-values. Abbreviations: LEAN – healthy control; P1 – diabetic control; P2 – metformin-treated diabetic rats.

m/z	**Annotation**	**Tentative compound /fragment**	**Trend vs P1**	**Mean LEAN**	**meanP1**	**mean P2**	**$\log_2$FC LEAN vs P1**	**$\log_2$FC P2 vs P1**	***q* value**
38.016	Low-mass organic fragment	–	Up	0.00360	0.00322	0.00360	0.16	0.16	0.0026
43.020	Low-mass fragment ($C_2H_3O^-$)	Fatty acid/ketone fragment	Mixed	0.00372	0.00510	0.00511	–0.46	0.00	0.0013
58.007	$C_3H_6O^-$ fragment	Small lipid/alcohol fragment	Mixed	0.00610	0.00840	0.00931	–0.46	0.15	2.97E–05
59.015	L-Asparagine fragment	Amino acid	Down	0.00568	0.00904	0.00873	–0.67	–0.05	7.47E–05
71.019	Hydrocarbon fragment	Lipid chain fragment	Mixed	0.0177	0.0222	0.0225	–0.33	0.02	3.39E–04
78.960	Hydrocarbon fragment	Alkene fragment	Mixed	0.130	0.093	0.0897	0.48	–0.05	3.67E–04
96.976	$C_4H_8O_3^-$	Fatty acid fragment	Mixed	0.0352	0.0305	0.0302	0.21	–0.01	8.24E–04
109.981	$CH_2OH–COO^-$ fragment	Fatty acid/alcohol	Up	0.00530	0.00456	0.00459	0.22	0.01	3.92E–03
113.065	$C_6H_9O_2^-$	Fatty acid fragment	Mixed	0.00183	0.00218	0.00236	–0.26	0.11	7.47E–05
115.011	Aspartic acid [M–H]⁻	Amino acid	Down	0.00032	0.00052	0.00047	–0.71	–0.14	9.44E–04
122.996	$C_5H_8NO_2^-$	Small nitrogen fragment	Up	0.00766	0.00659	0.00713	0.22	0.11	6.70E–03
127.080	$C_4H_7O_4^-$	Organic acid fragment	Mixed	0.00140	0.00186	0.00220	–0.41	0.24	1.26E–03
140.025	PE fragment ($C_2H_6NO_4P^-$)	Phosphatidylethanolamine	Mixed	0.00251	0.00215	0.00185	0.23	–0.22	1.06E–03
141.092	FA fragment ($C_{10}H_{13}O^-$)	Fatty acid	Mixed	0.00090	0.00108	0.00125	–0.26	0.22	7.47E–05
155.111	FA fragment ($C_{10}H_{15}O^-$)	Unsaturated FA fragment	Mixed	0.00088	0.00099	0.00116	–0.16	0.23	7.47E–05
169.126	FA fragment ($C_{11}H_{17}O^-$)	Fatty acid	Mixed	0.00066	0.00081	0.00099	–0.29	0.29	3.67E–04
183.141	FA fragment ($C_{12}H_{19}O^-$)	Fatty acid	Mixed	0.00044	0.00056	0.00072	–0.34	0.37	1.90E–04
197.161	FA fragment ($C_{13}H_{21}O^-$)	Fatty acid	Mixed	0.00025	0.00029	0.00038	–0.22	0.41	5.58E–04
211.175	FA fragment ($C_{14}H_{23}O^-$)	Fatty acid	Mixed	0.00024	0.00028	0.00038	–0.22	0.44	1.90E–04
221.059	FA fragment ($C_{14}H_{21}O_3^-$)	Myristic acid fragment	Down	0.00036	0.00057	0.00053	–0.66	–0.09	7.08E–06
225.004	FA fragment ($C_{14}H_{19}O_3^-$)	Hydroxylated FA	Up	0.00027	0.00023	0.00026	0.21	0.13	4.23E–02
225.190	FA fragment ($C_{14}H_{25}O_3^-$)	Fatty acid	Mixed	0.00021	0.00027	0.00041	–0.36	0.57	2.40E–04
227.205	FA fragment ($C_{14}H_{27}O_3^-$)	Fatty acid	Mixed	0.00010	0.00033	0.00069	–1.71	1.08	2.03E–04
237.221	FA fragment ($C_{15}H_{29}O_3^-$)	Fatty acid	Mixed	0.00020	0.00068	0.00118	–1.76	0.80	2.40E–04

241.043	FA fragment ($C_{16}H_{25}O_2^-$)	Palmitoleic acid fragment	Up	0.00128	0.00091	0.00092	0.50	0.03	1.55E–05
251.209	FA fragment	Fatty acid	Mixed	0.00012	0.00029	0.00047	–1.27	0.71	1.26E–03
255.238	FA fragment ($C_{16}H_{31}O_2^-$)	Palmitic acid $[M–H]^-$	Mixed	0.00536	0.0109	0.0166	–1.02	0.60	1.44E–04
256.240	FA fragment	Fatty acid	Mixed	0.00093	0.00191	0.00295	–1.04	0.63	1.44E–04
265.250	FA fragment ($C_{17}H_{33}O_2^-$)	Heptadecanoic acid	Mixed	0.00012	0.00022	0.00031	–0.79	0.51	2.59E–03
281.253	FA ($C_{18}H_{33}O_2^-$)	Oleic/linoleic acid $[M–H]^-$	Mixed	0.00285	0.00957	0.0137	–1.75	0.52	2.59E–03
283.270	FA ($C_{18}H_{35}O_2^-$)	Stearic acid $[M–H]^-$	Up	0.00435	0.00421	0.00482	0.05	0.20	8.19E–03
390.284	PA/PG fragment	Phospholipid backbone	Up	3.34E–05	2.97E–05	3.54E–05	0.17	0.25	3.37E–02
402.267	PA/PG fragment	Phospholipid	Up	4.03E–05	3.73E–05	4.48E–05	0.11	0.26	3.70E–02
404.288	PA/PG fragment	Phospholipid	Up	4.32E–05	4.15E–05	5.08E–05	0.06	0.29	1.33E–02
429.380	FA dimer / DAG fragment	Diacylglycerol fragment	Mixed	9.47E–05	1.72E–04	1.95E–04	–0.86	0.19	7.26E–03
447.350	FA/PL fragment	Lipid fragment	Mixed	4.51E–05	4.85E–05	5.51E–05	–0.10	0.19	2.12E–02
465.318	PA fragment	Phosphatidic acid	Down	9.14E–05	1.36E–04	1.06E–04	–0.57	–0.35	6.33E–03
536.376	PI fragment	Phosphatidylinositol	Up	3.93E–05	1.76E–05	2.07E–05	1.16	0.24	1.07E–08
634.477	PI fragment	Phosphatidylinositol	Mixed	1.08E–05	1.34E–05	1.51E–05	–0.32	0.17	3.67E–04
805.749	PS/PI molecular ion	Glycerophospholipid	Mixed	2.97E–06	5.89E–06	1.87E–05	–0.99	1.67	2.51E–03
806.840	PS/PI adduct	Glycerophospholipid	Mixed	3.29E–06	5.00E–06	1.24E–05	–0.61	1.31	2.59E–03

Table S2. Significant amino acid–related ions in negative-ion mode.

m/z	Mean LEAN	Mean P1	Mean P2	log_2FC (LEAN/P1)	log_2FC (P2/P1)	*p* value	*q* value	Trend vs P1	FDR sig
**40.006**	5.29×10^{-4}	5.85×10^{-4}	6.59×10^{-4}	−0.145	0.172	0.00186	0.01175	Mixed_vs_P1	TRUE
**42.002**	9.74×10^{-3}	1.16×10^{-2}	1.23×10^{-2}	−0.253	0.0871	0.00818	0.02742	Mixed_vs_P1	TRUE
**44.017**	5.46×10^{-4}	5.71×10^{-4}	6.79×10^{-4}	−0.0661	0.250	0.00338	0.01636	Mixed_vs_P1	TRUE
**49.008**	6.27×10^{-4}	7.57×10^{-4}	5.90×10^{-4}	−0.271	−0.359	0.01648	0.04696	Down_vs_P1	TRUE
**65.014**	4.52×10^{-2}	3.90×10^{-2}	3.86×10^{-2}	0.214	−0.0151	0.00345	0.01636	Mixed_vs_P1	TRUE
**73.023**	1.59×10^{-3}	1.75×10^{-3}	1.86×10^{-3}	−0.136	0.0826	0.01320	0.04179	Mixed_vs_P1	TRUE
**86.027**	2.36×10^{-3}	2.89×10^{-3}	2.90×10^{-3}	−0.293	0.0057	0.00210	0.01199	Mixed_vs_P1	TRUE
**87.010**	1.17×10^{-3}	1.31×10^{-3}	1.41×10^{-3}	−0.164	0.107	0.00703	0.02504	Mixed_vs_P1	TRUE
**89.025**	2.38×10^{-3}	3.99×10^{-3}	3.67×10^{-3}	−0.746	−0.120	0.00046	0.00441	Down_vs_P1	TRUE

**93.046**	7.40×10^{-4}	1.07×10^{-3}	9.13×10^{-4}	−0.532	−0.228	0.00026	0.00299	Down_vs_P1	TRUE
**114.027**	9.73×10^{-4}	1.60×10^{-3}	1.41×10^{-3}	−0.717	−0.180	0.00018	0.00267	Down_vs_P1	TRUE
**116.064**	3.37×10^{-4}	3.65×10^{-4}	4.04×10^{-4}	−0.112	0.148	0.00417	0.01748	Mixed_vs_P1	TRUE
**122.008**	5.33×10^{-4}	7.34×10^{-4}	6.57×10^{-4}	−0.462	−0.159	0.00623	0.02366	Down_vs_P1	TRUE
**128.067**	2.46×10^{-3}	2.24×10^{-3}	1.83×10^{-3}	0.136	−0.294	0.00059	0.00482	Mixed_vs_P1	TRUE
**153.017**	2.89×10^{-4}	3.23×10^{-4}	3.79×10^{-4}	−0.161	0.232	0.00019	0.00267	Mixed_vs_P1	TRUE
**164.071**	4.30×10^{-3}	3.09×10^{-3}	3.37×10^{-3}	0.476	0.124	2.41×10^{-7}	6.87×10^{-6}	Up_vs_P1	TRUE
**180.051**	1.45×10^{-3}	7.81×10^{-4}	8.54×10^{-4}	0.890	0.128	6.24×10^{-8}	3.56×10^{-6}	Up_vs_P1	TRUE
**209.033**	1.83×10^{-3}	1.70×10^{-3}	1.34×10^{-3}	0.113	−0.345	0.00149	0.01058	Mixed_vs_P1	TRUE
**235.194**	2.37×10^{-4}	2.25×10^{-4}	3.50×10^{-4}	0.0779	0.640	0.00429	0.01748	Up_vs_P1	TRUE
